# A pilot cross-sectional study of non-communicable diseases in TB household contacts

**DOI:** 10.5588/ijtldopen.23.0579

**Published:** 2024-04-01

**Authors:** Y. Hamada, A. Lugendo, T. Ntshiqa, G. Kubeka, J.M. Lalashowi, S. Mwastaula, K. Ntshamane, I. Sabi, S. Wilson, A. Copas, K. Velen, N.E. Ntinginya, L.T. Minja, I. Abubakar, S. Charalambous, M.X. Rangaka

**Affiliations:** ^1^ Institute for Global Health, University College London, London, UK;; ^2^ Mbeya Medical Research Centre, National Institute for Medical Research, Mbeya, United Republic of Tanzania;; ^3^ Implementation Research Division, The Aurum Institute, Johannesburg,; ^4^ School of Public Health, University of the Witwatersrand, Johannesburg,; ^5^ School of Public Health, and Clinical Infectious Disease Research Institute-Africa, University of Cape Town, Cape Town, South Africa

**Keywords:** NCD, screening, epidemiology, HHCs, integrated NCD screening and care

## Abstract

**BACKGROUND:**

Data on the prevalence of non-communicable diseases (NCDs) in TB household contacts (HHCs) are limited, yet important to inform integrated screening and care for NCD within contact investigations. It is also unclear if screening these contacts reveals more people with NCDs than individuals in the same neighbourhood.

**METHOD:**

We conducted a pilot cross-sectional study in South Africa and Tanzania, enrolling adult HHCs of TB and individuals in neighbourhood households (controls). We inquired about known NCD and systematically measured blood pressure, and tested for spot blood glucose and haemoglobin A1c.

**RESULTS:**

We enrolled 203 adult contacts of 111 persons with TB and 160 controls. Among contacts, respectively 12.2% (95% CI 8.3–17.6) and 39.7% (95% CI 33.1–46.7) had diabetes and hypertension, compared to 14.1% (95% CI 9.2–21.0) and 44.7% (95% CI 36.9–52.7) among controls. More than half of NCDs were newly identified. We did not find a significant difference in the prevalence of at least one NCD between the two groups (OR 0.85, 95% CI 0.50–1.45, adjusted for age and sex).

**CONCLUSIONS:**

We found a high prevalence of undiagnosed NCDs among contacts, suggesting a potential benefit of integrating NCD screening and care within contact investigations. Screening in the same community might similarly find undiagnosed NCDs.

## RÉSUMÉ

**CONTEXTE:**

Les données sur la prévalence des maladies non transmissibles (NCD, pour l’anglais « *non-communicable diseases* ») chez les contacts familiaux (HHC, pour l’anglais « *household contacts* ») de personnes atteintes de TB sont restreintes, mais elles revêtent une grande importance pour le dépistage et la prise en charge intégrée des NCD dans le cadre des enquêtes sur les contacts. De plus, on ignore si le dépistage de ces contacts permet de détecter davantage de personnes atteintes de NCD par rapport aux les individus résidant dans le même quartier.

**MÉTHODE:**

Nous avons réalisé une étude pilote transversale en Afrique du Sud et en Tanzanie, au cours de laquelle nous avons recruté des adultes HHC de personnes atteintes de TB et des individus vivant dans les ménages voisins (témoins). Nous les avons interrogés sur les NCD connues et avons systématiquement mesuré la pression artérielle, ainsi que réalisé des tests de de glycémie et d’hémoglobine glyquée.

**RÉSULTATS:**

Un total de 203 contacts adultes de 111 personnes atteintes de TB et 160 témoins ont été répertoriés. Parmi ces contacts, respectivement 12,2% (IC à 95% 8,3–17,6) et 39,7% (IC à 95% 33,1–46,7) souffraient de diabète et d'hypertension, contre 14,1% (IC à 95% 9,2–21,0) et 44,7% (IC à 95% 36,9–52,7) chez les témoins. Plus de la moitié des NCD ont été récemment découvertes. Aucune disparité significative n'a été observée dans la prévalence d'au moins une NCD entre les deux groupes (OR 0,85 ; 95% CI 0,50–1,45, ajusté pour l'âge et le sexe).

**CONCLUSIONS:**

Nous avons observé une fréquence élevée de NCDs non diagnostiquées parmi les contacts, ce qui indique qu'il pourrait être potentiellement bénéfique d'inclure le dépistage et les soins des NCD dans les enquêtes sur les contacts. Le dépistage au sein de la même communauté pourrait également révéler des NCD non diagnostiquées.

Non-communicable diseases (NCDs) present a formidable challenge to ending the TB epidemic. Diabetes mellitus (DM), tobacco smoking and disorders due to alcohol use are among the key risk factors for TB, alongside HIV and undernutrition.^1^ The burden of NCDs, such as DM and cardiovascular diseases (CVD), is rising in low- and middle-income countries (LMICs), including South Africa and Tanzania. For example, from 2011 to 2021, the age-adjusted prevalence of DM increased from 7.0% to 10.8% in South Africa and 2.8% to 12.3% in Tanzania.^2^ The convergence of NCDs and TB, coupled with HIV, has created a challenging syndemic with potentially far-reaching health and economic consequences. In LMICs, shared risk factors often linked to poverty lead to the co-occurrence of multiple diseases – a condition called multimorbidity (defined as the co-existence of two or more chronic conditions).^3^ There is a need for integrated patient-centric prevention and care models to address multimorbidity effectively.^3^

The WHO has recommended bidirectional screening for TB and DM.^4^ Experts have also recommended screening for other NCDs among people with TB.^5^ However, such recommendations have not extended to household contacts (HHCs) of people with TB. HHCs are likely to share predispositions to NCDs, such as smoking, alcohol use and poverty, and the presence of NCDs, if untreated, can increase their risk for TB development. Data among the general population suggest that NCDs cluster in households.^6–8^ Contact tracing for TB is an established intervention targeting households.^9^ Integrating NCD screening, care and prevention within TB contact tracing can leverage resources and potentially enhance its value for money. Furthermore, given the shared risk factors among household members, HHCs may have a higher prevalence of NCDs than the general population, which would strengthen the rationale for targeting HHCs for NCD screening; however, the evidence is sparse. Previous studies have explored the prevalence of NCDs, mainly DM, among HHCs. However, these studies lacked control groups, limiting their ability to provide comparative data.^10–13^ In the absence of a control group, understanding the comparative prevalence of NCDs in contacts has been challenging. A comparison with national prevalence provides limited insights as NCD prevalence can vary regionally, and demographics may differ between HHCs and the general population. Furthermore, data on other NCDs, such as hypertension and chronic kidney disease (CKD), were limited in previous studies.

We investigated the prevalence of NCDs, the pattern of NCD overlap and multimorbidity among HHCs of TB patients through systematic screening. We also compared them with individuals in the same geographical areas.

## METHOD

### Study design and participants

This is a pilot cross-sectional study that assessed the prevalence of NCDs among HHCs of people with TB compared to individuals in the same neighbourhood as a control group. This study was nested within Phase 1 of the Community and Universal Testing for TB among contacts (CUT-TB) study, and was conducted in Ekurhuleni in South Africa and the Mbeya Region in Tanzania. Annual TB notification rates in these areas are around 300 and 150 cases per 100,000 populations, respectively.^14,15^ There is a lack of local data on NCD prevalence. In Gauteng, where Ekrhuleni is located, the 2016 Demographic and Health Survey (DHS) reported a hypertension prevalence of 42.3% in women and 39.5% in men, and a DM prevalence of 9.3% in women and 6.6% in men.^16^ In the Mbeya Region, the 2022 DHS reported a hypertension prevalence of 6.4% in women and 9.7% in men.^17^

We consecutively enrolled index patients diagnosed with bacteriologically confirmed drug-susceptible or drug-resistant TB of all ages who did not live alone from clinics in the study sites. After obtaining consent, our field team visited their households to enrol adult HHCs (≥18 years). For controls, we enrolled one neighbourhood household per TB patient, and their adult household members were invited. In South Africa, we generated random coordinates using the ‘*sf*’ R package (R Computing, Vienna, Austria) to identify households within the same ward as the TB patients.^18^ The field team visited these households, and after obtaining consent from the household heads, we invited their household members to participate in the study. If declined, we repeated the above process until at least one household per TB patient was enrolled. In Tanzania, we did the same in the city of Mbeya, but in the other areas, we enrolled households closest to the index households due to operational challenges.

### Study procedures

A team, including research assistants and a nurse, collected the data. At baseline, we collected sociodemographic information (e.g., age, sex, years of education and employment status), risk factors such as smoking and alcohol use and medical history through interviews. We also measured height, weight and blood pressure. For blood pressure, two readings were performed, and the second reading was used. We tested for random blood glucose (RBG) using serum samples in South Africa and capillary blood in Tanzania. Regardless of RBG results, all participants were also tested for haemoglobin A1c (HbA1c), total cholesterol and creatinine using venous blood in local laboratories. Participants who were found to have NCD were referred to nearby clinics. HIV counselling and testing was offered if their status was unknown for more than 6 months. All participants were asked to sign the consent form before HIV testing was done. In addition, a trained counsellor conducted HIV counselling before and after the HIV test in a private room.

### Outcomes

We defined DM as HbA1c ≥6.5%, or history of diagnosis.^19^ Hypertension was defined as systolic blood pressure ≥140 mmHg and/or diastolic blood pressure ≥90 mmHg, or history of diagnosis.^19^ CKD was defined as an estimated glomerular filtration rate (eGFR) <60 mL/min, which was calculated using the 2021 CKD Epidemiology Collaboration creatinine equation.^20,21^ For ascertaining NCD, we did not require multiple measurements; hence, our investigation was meant for screening rather than confirmatory diagnosis. A single measurement has been pragmatically used in other epidemiological studies evaluating NCD prevalence.^11,12,22^ The primary outcome was the presence of at least one of the NCDs above. As secondary outcomes, we investigated the prevalence of individual NCD as well as multimorbidity involving two or more conditions of NCDs and HIV. Additionally, we calculated CVD risk over 10 years using the WHO CVD laboratory-based risk chart (Supplementary Figure S1), and evaluated the prevalence of CVD risk >20% that requires statin therapy as per the WHO guidelines.^23^

### Sample size

We planned to enrol 100 households of index TB patients and 100 neighbourhood households in each country. We assumed that respectively 200 and 300 adult HHCs and respectively 250 and 350 neighbourhood controls would be enrolled in South Africa and Tanzania. Based on existing literature, the prevalence of NCDs was expected to range from 8% (e.g., DM) to 40% (hypertension). Assuming a conservative intra-cluster correlation coefficient of 0.1 for household clustering, we estimated that the prevalence of NCDs in each country could be estimated with a margin of error at <5–7.5% (Table 1A). Furthermore, assuming that the prevalence of at least one NCD was 40% among neighbourhood controls, we used a normal approximation method to calculate a power to detect a difference in the prevalence,^24^ ignoring the clustering as the association can be estimated within clusters. It was estimated that enrolling at least 200 HHCs and 250 neighbourhood controls would provide 80% power to detect at least 33% higher prevalence (i.e., the prevalence of ≥53.2%) in HHCs at a 5% significance level.

**Table 1.** Characteristics of study participants.*

	Household contacts	Neighbourhood household members
	(*n* = 203)	(*n* = 160)
	*n/N* (%)	*n/N* (%)
Country		
South Africa	156 (84.4)	135 (76.8)
Tanzania	47 (15.6)	25 (23.2)
Age, years, median [IQR]	40.0 [30.0–59.0]	42.0 [31.0–56.0]
Female	135/203 (66.5)	80/160 (50.0)
Current smoker	33/203 (16.3)	42/160 (26.2)
Alcohol use	78/203 (38.4)	86/160 (53.8)
Obesity (BMI ≥ 30 kg/m^2^)	35/201 (17.4)	18/160 (11.2)
BMI, kg/m^2^, median [IQR]	24.2 [20.4–28.1]	21.3 [18.3–25.7]
Known HIV-positive status	29/203 (14.3)	21/160 (13.1)
Time spent on education, years, median [IQR]	10 [7–12]	11 [8–12]
Employment		
Employed	25 (12.3)	12 (7.5)
Self-employed	44 (21.7)	30 (18.8)
Unemployed	119 (58.6)	108 (67.5)
Others	15 (7.4)	10 (6.2)

* Denominators vary because of missing data.

IQR = interquartile range; BMI = body mass index.

### Statistical analysis

We presented the prevalence of NCDs, multimorbidity and CVD risk >20% in HHCs and in neighbourhood contacts with robust 95% confidence intervals acknowledging clustering within households. To calculate the prevalence, we excluded participants with missing NCD data.

To determine whether HHCs have a higher likelihood of NCD than neighbourhood controls of the same age and sex, we calculated the odds ratios for NCDs in contacts vs. controls. This was achieved using logistic regression models fitted with a generalised estimating equation, accounting for clustering by index cases. The model adjusted for the pre-specified variables of age and sex. This analysis was conducted for the outcomes defined above. Although our initial plan was to analyse each country separately, we ultimately merged the two populations due to the limited sample size from Tanzania, and as a result, the model was not adjusted for countries. We presented the pattern of multimorbidity visually.

### Ethical considerations

We obtained written informed consent from all study participants. Ethical approval was obtained from the Ethics Committee at the University of the Witwatersrand, Johannesburg, South Africa; the National Institute for Medical Research, Mbeya, United Republic of Tanzania; and University College London, London, UK.

## RESULTS

### Characteristics of participants

**Figure 1.**
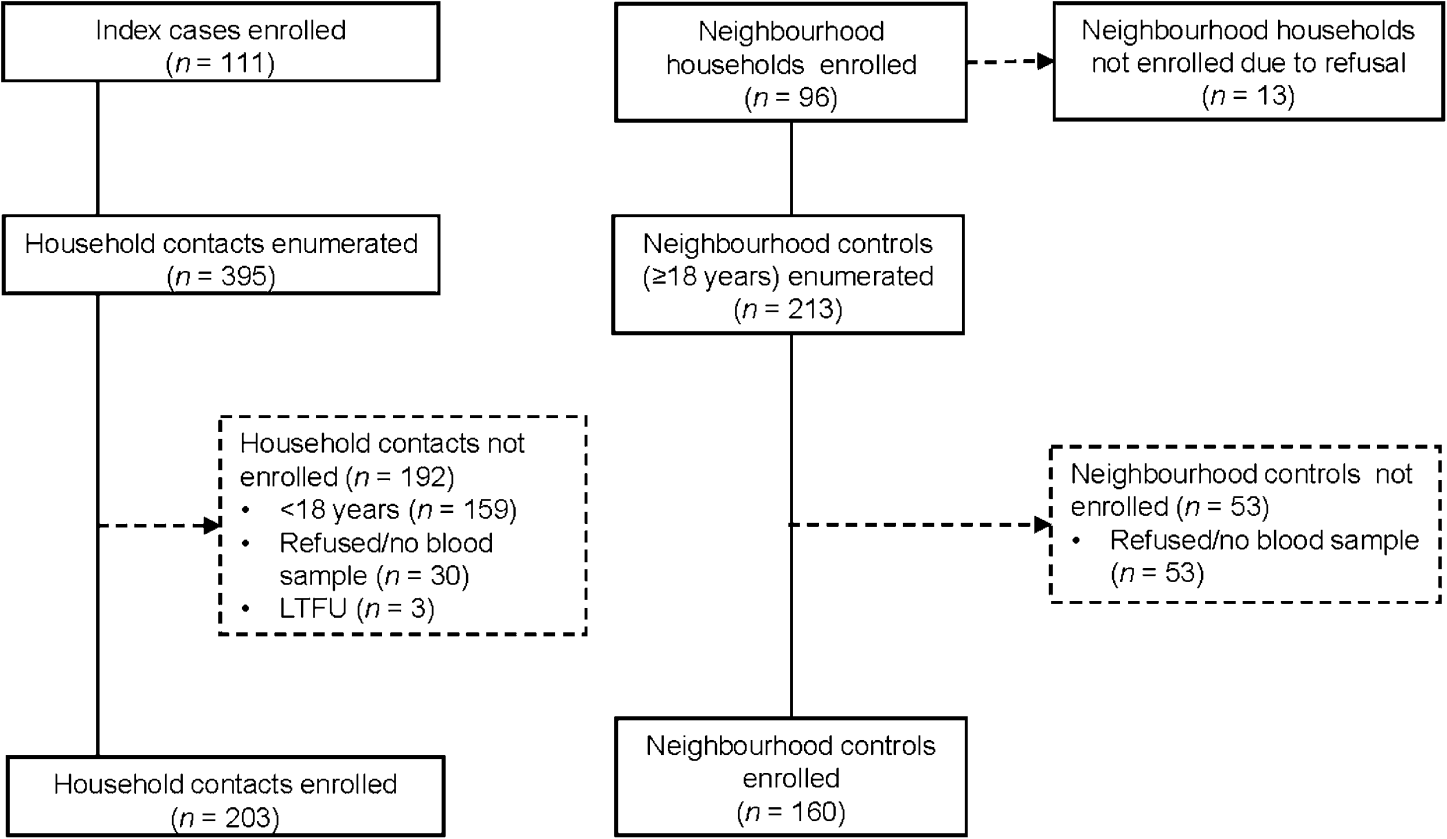
Enrolment of participants.

In total, we enrolled 203 adult HHCs of 111 persons with TB. The majority of these contacts (76.8%, 156/203) were from South Africa, linked to 81 persons with TB (Figure 1). In addition, we enrolled 135 adults from 81 neighbourhood households in South Africa. In Tanzania, we could not enrol the same number of neighbourhood households as the households of index TB cases (*n* = 30) due to refusal; as a result, we enrolled 25 adults from 17 neighbourhood households. Among the 17 households, one was enrolled in the City of Mbeya. Overall, 16 out of 98 (16.3%) neighbourhood households were enrolled from rural sites in Tanzania through non-random sampling.

Table 1 presents the participant characteristics of the combined cohort. The median age was 40.0 among contacts and 42.0 years among controls, respectively. There were more females among HHCs than in neighbourhood households (66.5% vs. 50.0%). Characteristics by country are presented in Supplementary Table S2.

### Prevalence of non-communicable disease and multimorbidity

Among HHCs, 23 had DM (12.2%, 95% confidence interval [CI] 8.3–17.6), of whom 17 (73.9%) were newly identified (Table 2). Hypertension was present in 39.2% of contacts, and more than half (55.7%, 44/79) were newly identified, and 10.0% (19/190) had CKD. Overall, at least one NCD was present in 49.5% of contacts. The proportion of individuals with >20% risk for developing CVDs was around 3% in both contacts and controls (3.2%, 95% CI 1.4–7.0 vs. 4.0%, 95% CI 1.8–8.5). When stratified by country, the prevalence of DM among contacts was 11.8% in South Africa and 13.3% in Tanzania, and the prevalence of hypertension was 40.8% and 34.0%, respectively (Supplementary Table S2).

**Table 2.** Prevalence of NCD among household contacts and neighbourhood controls.

Variable	Household contacts		Controls		Household contacts vs. controls	*P* value
	*n/N*	% (95% CI)	*n/N*	% (95% CI)	aOR* (95% CI)	
DM	23/189	12.2 (8.3–17.6)	21/149	14.1 (9.2–21.0)	0.73 (0.37–1.46)	0.38
Newly identified DM	17/189	9 (5.7–14)	18/152	11.8 (7.4–18.4)	0.65 (0.32–1.35)	0.25
Hypertension	79/199	39.7 (33.1–46.7)	71/159	44.7 (36.9–52.7)	0.68 (0.4–1.14)	0.14
Newly identified hypertension	44/199	22.1 (16.9–28.4)	37/159	23.3 (17.2–30.6)	0.9 (0.53–1.52)	0.68
CKD	19/190	10 (6.5–15.2)	14/157	8.9 (5.3–14.5)	1.65 (0.7–3.9)	0.25
CVD risk ≥20% over 10 years	6/187	3.2 (1.4–7.0)	6/151	4.0 (1.8–8.5)	0.72 (0.18–2.91)	0.65
At least one NCD	93/190	49.5 (42.4–56.5)	79/153	51.6 (43.5–59.7)	0.85 (0.50–1.45)	0.56
Multimorbidity^†^	34/203	16.7 (12.2–22.6)	33/160	20.6 (14.9–27.8)	0.83 (0.46–1.51)	0.54
Current smoker	33/203	16.3 (11.7–22.2)	42/160	26.2 (19.8–33.9)	0.84 (0.47–1.52)	0.57
Alcohol use	78/203	38.4 (31.9–45.3)	86/160	53.8 (45.8–61.5)	0.6 (0.38–0.95)	0.03
Obesity	35/201	17.4 (12.8–23.3)	18/160	11.2 (7.2–17.2)	1.41 (0.73–2.73)	0.3

* Adjusted for age and sex.

† Two or more conditions of DM, hypertension, CKD and HIV.

NCD = non-communicable disease; CI = confidence interval; aOR = adjusted odds ratio; DM = diabetes mellitus; CKD = chronic kidney disease; CVD = cardiovascular disease.

When compared with neighbourhood controls, the prevalence of at least one NCD was similar (49.5%, 95% CI 42.4–56.5 vs. 51.6%, 95% CI 43.5–59.7). When adjusted for age and sex, HHCs did not have a higher likelihood of having at least one NCD than neighbourhood controls (odds ratio [OR] 0.85, 95% CI 0.50–1.45). Likewise, the prevalence of individual NCDs was similar between contacts and neighbourhood controls overall (Table 2) and in South Africa, but perhaps higher among controls in Tanzania (Supplementary Table S2).

**Figure 2.**
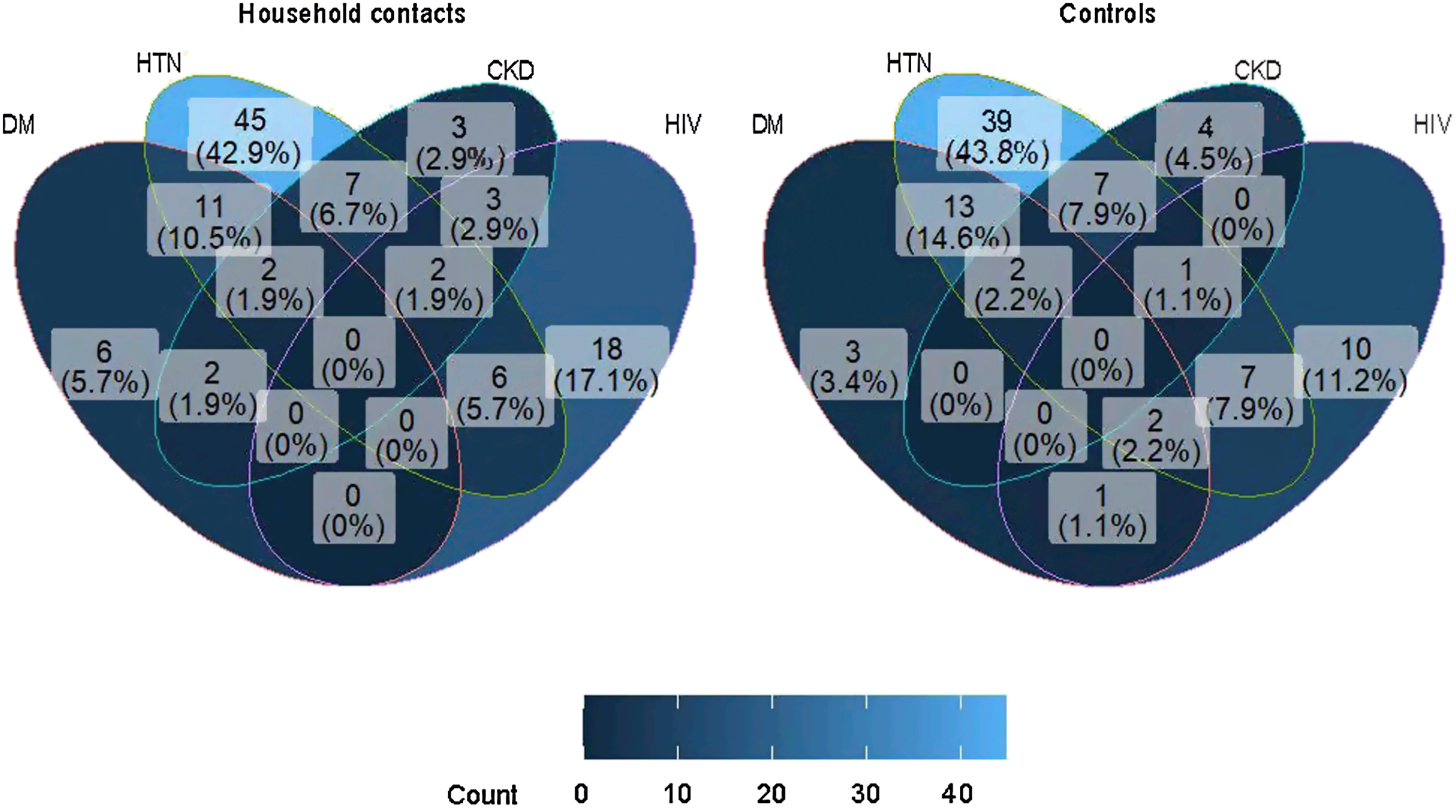
The pattern of multimorbidity among household contacts and controls. DM = diabetes mellitus; HTN = hypertension; CKD = chronic kidney disease.

Among household contacts, 16.7% (95% CI 12.2–22.6) had multimorbidity, comprising at least two conditions out of NCD and HIV, compared to 20.6% (95% CI 14.9–27.8) among controls. Figure 2 presents the pattern of NCD overlap and multimorbidity. DM and hypertension were most commonly overlapping; among 34 contacts with multimorbidity, 13 (38.2%) had both DM and hypertension. A similar pattern was observed in neighbourhood controls.

## DISCUSSION

Our pilot study found a high prevalence of NCD, most of which were undiagnosed prior to the present study. The prevalence of DM was 12.2% among contacts, and around 70% of them were newly diagnosed. This suggests that integrating screening for NCD within contact investigations would help identify those who are otherwise unaware of their NCD. Furthermore, people with DM are at an increased risk for TB, especially if their glycaemic levels are poorly controlled.^25,26^ Thus, early identification and treatment of contacts with DM might help reduce TB incidence. A recent cluster randomised controlled trial (RCT) demonstrated the effect of nutritional supplementation to prevent TB among HHCs.^27^ However, the impact of an integrated care approach addressing TB, DM and other NCDs remains unclear. To evaluate the effectiveness of this integrated programme, a similar RCT is needed. Importantly, the outcomes of this trial should extend beyond TB incidence, capturing the broader health implications, including NCD outcomes. This will provide a more complete picture of an integrated healthcare strategy.

Contrary to previous studies,^10–13^ our study included a control group of household members from randomly selected neighbourhood households and accounted for demographic differences. We found a similarly high prevalence of NCD both in HHCs and neighbourhood controls. Therefore, screening for NCD might warrant extension to people in the same community. Depending on the feasibility, multiple options could be considered. For example, contact investigations could involve neighbourhood households for both TB and NCD screening. The WHO recommends systematic screening for TB disease among the general population in areas with a high TB prevalence.^28^ NCD screening could be integrated into such community-wide TB screening activities. Of note, due to the low statistical power of our study, we cannot rule out a difference in the NCD prevalence between contacts and neighbourhood controls.

No study to date has evaluated multimorbidity among contacts of TB. Consistent with our prior knowledge, DM and hypertension, known to increase CVD risk, overlapped most commonly.^29^ In our cohort, 3.2% of contacts had a ≥20% risk of developing CVD within 10 years, warranting statin therapy.^30^ The risk may be heightened by HIV co-infection^31^ and by TB.^32^ The WHO recommends assessment of CVD risk in individuals at risk for CVD, such as people aged >40 years and smokers. It is a missed opportunity not to conduct CVD risk assessment in contacts who have these conditions to prevent CVD.

There are limitations in our study. First, we could not enrol the target number of households due to the delay in the initiation of this pilot study, coupled with a faster recruitment into the parent study. Furthermore, the number of participants per household was smaller than expected, especially among neighbourhood control, because of unavailability at the time of the household visit and lack of motivation for NCD screening. It may be possible that people who were at risk for NCD were overrepresented. In addition, integrated TB and NCD screening may also face low participation rates, and strategies to promote participation are needed to maximise the cost-effectiveness and impact of the screening. Second, in rural Tanzanian sites, operational challenges prevented the random selection of control households. The median age was higher (49 years) than contacts (37 years), and around 50% had DM. This may have increased the participation of older people and those with comorbidity, probably influenced by availability and willingness to participate in the study. Nonetheless, the results are similar when restricted to South Africa. Third, the ascertainment of NCD, including hypertension, was based on measurement on a single day. Therefore, the NCD prevalence might have been overestimated.^33^ Fourth, we did not evaluate the subsequent treatment and control of NCD identified. Inadequate follow-up and management of NCDs could diminish the effectiveness of our screening activities.

## CONCLUSION

In summary, our pilot study highlights a high prevalence of undiagnosed NCDs, particularly DM, among contacts of TB patients and individuals in the same communities. However, our small sample size precluded demonstrating a difference in the NCD prevalence. A larger study is warranted to determine whether NCD screening in contacts leads to higher yields. Nonetheless, the high prevalence of undiagnosed NCDs underscores the potential benefits of NCD screening building on existing TB contact investigations. Future trials should evaluate the comprehensive health benefits of such integrated care among contacts. Furthermore, comparable NCD prevalence observed in individuals from the same neighbourhoods as TB contacts suggests a potential rationale for expanding NCD screening to encompass the wider community. Further studies are needed to evaluate the effectiveness, feasibility and acceptability of these expanded, integrated TB and NCD screening programmes.

## Supplementary Material


