# Supplementary material for: A pilot cross-sectional study of non-communicable diseases in TB household contacts
Source: IJTLD Open. 2024 Apr 1;1(4):154–9. doi: 10.5588/ijtldopen.23.0579 (PMC11231826; doi:10.5588/ijtldopen.23.0579)
Supplement: Supplementary file 1 [file iutld_ijtld_open_23.0579_supplementarydata1.pdf]

http://dx.doi.org/10.5588/ijtldopen.23.0579

A pilot cross-sectional study of non-communicable diseases in TB household contacts

SUPPLEMENTARY DATA

Supplementary Figure S1. World Health Organization cardiovascular disease risk laboratory based charts

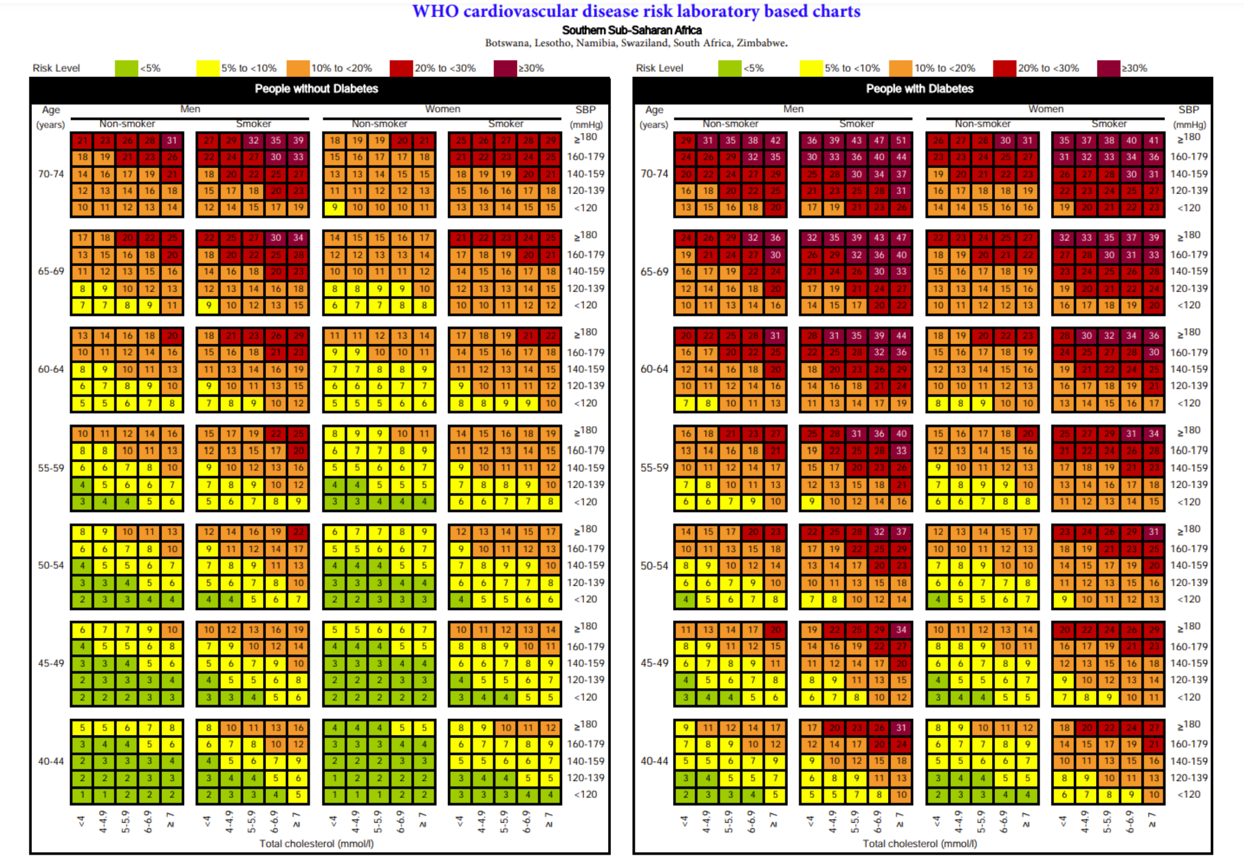

WHO cardiovascular disease risk laboratory-based charts

Eastern Sub-Saharan Africa

Burundi, Comoros, Djibouti, Eritrea, Ethiopia, Kenya, Madagascar, Mozambique, Malawi, Rwanda, Somalia, Tanzania, Uganda, Zambia

Risk Level: <5% | 5% to <10% | 10% to <20% | 20% to <30% | ≥30%

People without Diabetes

Age (years)	SBP (mmHg)	Men Non-smoker					Men Smoker					Women Non-smoker					Women Smoker				
	Total cholesterol (mmol/l)	<4	4-4.9	5-5.9	6-6.9	≥ 7	<4	4-4.9	5-5.9	6-6.9	≥ 7	<4	4-4.9	5-5.9	6-6.9	≥ 7	<4	4-4.9	5-5.9	6-6.9	≥ 7
70-74	≥180	22	24	27	29	32	28	30	33	36	39	16	17	17	18	19	22	23	24	25	26
	160-179	18	20	22	24	26	23	25	28	30	33	14	14	15	15	16	19	20	20	21	22
	140-159	15	17	18	20	22	19	21	23	25	28	12	12	13	13	14	16	17	17	18	19
	120-139	12	14	15	16	18	16	17	19	21	23	10	10	11	11	12	14	14	15	15	16
	<120	10	11	12	14	15	13	14	16	17	19	8	9	9	9	10	12	12	12	13	14
65-69	≥180	18	19	21	23	26	23	26	28	31	34	12	13	14	14	15	19	19	20	21	23
	160-179	14	16	17	19	21	19	21	23	25	28	10	11	11	12	13	15	16	17	18	19
	140-159	11	12	14	15	17	15	17	19	21	23	9	9	9	10	10	13	13	14	15	16
	120-139	9	10	11	12	14	12	14	15	17	19	7	7	8	8	9	11	11	12	12	13
	<120	7	8	9	10	11	10	11	12	14	15	6	6	6	7	7	9	9	10	10	11
60-64	≥180	14	15	17	19	21	19	21	24	27	30	10	10	11	11	12	15	16	17	18	20
	160-179	11	12	13	15	17	15	17	19	21	24	8	8	9	9	10	13	13	14	15	16
	140-159	8	9	10	12	13	12	13	15	17	19	6	7	7	8	8	10	11	12	12	13
	120-139	7	7	8	9	10	9	11	12	14	15	5	5	6	6	7	8	9	9	10	11
	<120	5	6	6	7	8	7	8	9	11	12	4	4	5	5	5	7	7	8	8	9
55-59	≥180	11	12	13	15	17	16	18	20	23	26	7	8	8	9	10	13	14	15	16	17
	160-179	8	9	10	11	13	12	14	16	18	20	6	6	7	7	8	10	11	12	13	14
	140-159	6	7	8	9	10	10	11	12	14	16	5	5	5	6	6	8	9	9	10	11
	120-139	5	5	6	7	8	7	8	9	11	13	4	4	4	5	5	6	7	8	8	9
	<120	4	4	5	5	6	6	6	7	8	10	3	3	3	4	4	5	6	6	7	7
50-54	≥180	8	9	10	12	13	13	15	17	19	22	6	6	7	7	8	11	12	12	14	15
	160-179	6	7	8	9	10	10	11	13	15	17	4	5	5	6	6	8	9	10	11	12
	140-159	5	5	6	7	8	8	9	10	11	13	3	4	4	4	5	6	7	8	8	9
	120-139	3	4	4	5	6	6	6	7	9	10	3	3	3	3	4	5	5	6	7	7
	<120	3	3	3	4	5	4	5	6	7	8	2	2	2	3	3	4	4	5	5	6
45-49	≥180	6	7	8	9	11	11	12	14	16	19	4	5	5	6	6	9	10	11	12	13
	160-179	5	5	6	7	8	8	9	11	12	14	3	4	4	4	5	7	7	8	9	10
	140-159	3	4	4	5	6	6	7	8	9	11	2	3	3	3	4	5	6	6	7	8
	120-139	2	3	3	4	4	4	5	6	7	8	2	2	2	2	3	4	4	5	5	6
	<120	2	2	2	3	3	3	4	4	5	6	1	2	2	2	2	3	3	4	4	5
40-44	≥180	5	6	6	7	9	9	10	12	14	16	3	4	4	4	5	7	8	9	10	11
	160-179	3	4	4	5	6	6	7	9	10	12	2	3	3	3	4	5	6	7	8	8
	140-159	2	3	3	4	5	5	5	6	7	9	2	2	2	2	3	4	5	5	6	6
	120-139	2	2	2	3	3	3	4	5	5	7	1	1	2	2	2	3	3	4	4	5
	<120	1	1	2	2	2	2	3	3	4	5	1	1	1	1	2	2	3	3	3	4

People with Diabetes

Age (years)	SBP (mmHg)	Men Non-smoker					Men Smoker					Women Non-smoker					Women Smoker				
	Total cholesterol (mmol/l)	<4	4-4.9	5-5.9	6-6.9	≥ 7	<4	4-4.9	5-5.9	6-6.9	≥ 7	<4	4-4.9	5-5.9	6-6.9	≥ 7	<4	4-4.9	5-5.9	6-6.9	≥ 7
70-74	≥180	30	32	35	38	42	37	40	43	47	51	24	25	25	26	28	32	33	34	36	37
	160-179	25	27	29	32	35	31	34	37	40	43	20	21	22	23	24	27	28	30	31	32
	140-159	20	22	25	27	30	26	28	31	34	37	17	18	19	19	20	24	24	25	26	28
	120-139	17	19	20	23	25	21	23	26	28	31	15	15	16	16	17	20	21	22	23	24
	<120	14	15	17	19	21	18	19	22	24	26	12	13	13	14	15	17	18	19	19	20
65-69	≥180	25	27	30	33	36	33	36	39	43	47	20	21	22	23	24	29	30	31	33	34
	160-179	20	22	25	27	30	27	30	33	36	40	16	17	18	19	20	24	25	27	28	29
	140-159	16	18	20	22	25	22	24	27	30	33	14	14	15	16	17	20	21	22	24	25
	120-139	13	15	16	18	20	18	20	22	25	27	11	12	13	13	14	17	18	19	20	21
	<120	11	12	13	15	17	14	16	18	20	23	9	10	10	11	12	14	15	16	17	18
60-64	≥180	21	23	25	28	32	29	32	35	39	44	16	17	18	19	20	26	27	29	30	32
	160-179	16	18	20	23	26	23	26	29	32	36	13	14	15	16	17	21	22	24	25	27
	140-159	13	14	16	18	21	19	21	23	26	30	11	11	12	13	14	17	18	20	21	22
	120-139	10	11	13	15	17	15	17	19	21	24	9	9	10	11	11	14	15	16	17	19
	<120	8	9	10	12	13	12	13	15	17	19	7	8	8	9	9	12	12	13	14	15
55-59	≥180	17	19	22	24	27	26	29	32	36	40	13	14	15	16	18	23	24	26	28	30
	160-179	13	15	17	19	22	20	23	26	29	33	11	11	12	13	14	18	20	21	23	25
	140-159	10	12	13	15	17	16	18	20	23	26	8	9	10	11	11	15	16	17	19	20
	120-139	8	9	10	12	13	12	14	16	18	21	7	7	8	8	9	12	13	14	15	16
	<120	6	7	8	9	10	9	11	12	14	17	5	6	6	7	7	10	10	11	12	13
50-54	≥180	15	16	18	21	23	23	26	29	33	37	11	12	13	14	15	20	22	24	26	28
	160-179	11	12	14	16	18	18	20	23	26	30	9	9	10	11	12	16	17	19	21	23
	140-159	8	9	11	12	14	13	15	17	20	23	7	7	8	9	9	13	14	15	16	18
	120-139	6	7	8	9	11	10	12	13	16	18	5	6	6	7	7	10	11	12	13	14
	<120	5	5	6	7	8	8	9	10	12	14	4	4	5	5	6	8	9	9	10	12
45-49	≥180	12	14	15	17	20	21	23	26	30	34	9	10	11	12	13	18	20	22	24	26
	160-179	9	10	11	13	15	15	17	20	23	27	7	7	8	9	10	14	15	17	19	21
	140-159	6	7	8	10	12	11	13	15	18	21	5	6	6	7	8	11	12	13	15	16
	120-139	5	5	6	7	9	8	10	11	13	16	4	4	5	5	6	8	9	10	11	13
	<120	3	4	5	5	7	6	7	8	10	12	3	3	4	4	5	6	7	8	9	10
40-44	≥180	10	11	13	15	17	18	21	23	27	32	7	8	9	10	11	16	18	20	22	24
	160-179	7	8	9	11	13	13	15	17	20	24	5	6	7	7	8	12	13	15	17	19
	140-159	5	6	7	8	9	10	11	13	15	18	4	4	5	6	6	9	10	11	13	15
	120-139	4	4	5	6	7	7	8	9	11	14	3	3	4	4	5	7	8	9	10	11
	<120	3	3	4	4	5	5	6	7	8	10	2	3	3	3	4	5	6	7	8	9

Supplementary Table S1. The margin of error by prevalence of NCD and number of contacts

	Prevalence					
	8%	15%	20%	30%	40%	50%
# of contacts	Margin of error (%)					
200	4.3	5.4	6	6.9	7.3	7.5
250	3.8	5	5.5	6.3	6.7	6.8
300	3.6	4.6	5.1	5.9	6.2	6.4
350	3.3	4.4	4.8	5.5	5.9	6

The effective sample size (ESS) was calculated as ESS = mk / DE, where m is the cluster sample size (i.e., number of contacts per index), k is the number of clusters (i.e., 100 index cases), and DE is the design effect, calculated assuming an intra-cluster correlation coefficient of 0.1. Total number of contacts is calculated as mk. The 95%-confidence interval (CI) for an expected prevalence of Non-Communicable Diseases (NCD) was estimated using the Clopper–Pearson method, and the margin of error (± 95% CI) was presented.

Supplementary Table S2.Characteristics of participants by country

	South Africa		Tanzania	
	Household contacts (N = 156)	Neighbourhood household members (N = 135)	Household contacts (N = 47)	Neighbourhood household members (N = 25)
Age (median [IQR])	42.5 [30.0, 61.3]	40.00 [31.0, 54.5]	37.0 [29.5, 48.5]	49.0 [33.0, 60.0]
Female (%)	101 (64.7)	67 (49.6)	34 (72.3)	13 (52.0)
Current smoker	33 (21.2)	41 (30.4)	0 (0.0)	1 (4.0)
Alcohol use	62 (39.7)	76 (56.3)	16 (34.0)	10 (40.0)
Obesity (BMI ≥ 30 Kg/m^2)	25.1 [20.1, 28.6]	20.6 [17.9, 25.1]	23.1 [21.4, 25.2]	24.8 [22.0, 26.5]
BMI	31 (20.1)	16 (11.9)	4 (8.5)	2 (8.0)
Known HIV-positive status	25 (22.5)	17 (18.3)	4 (11.4)	4 (17.4)

IQR: interquartile range; BMI: body mass index; HIV: human immunodeficiency virus

Supplementary Table S3. Prevalence of NCD among household contacts and neighbourhood controls by country

	South Africa					Tanzania				
	Household contacts		Controls		Household contacts vs controls	Household contacts		Controls		Household contacts vs controls
Variable	n/N	% (95% CI)	n/N	% (95% CI)	OR* (95% CI), p value	n/N	% (95% CI)	n/N	% (95% CI)	OR* (95% CI), p value
Diabetes	17/144	11.8 (7.5-18.2)	10/126	7.9 (4.3-14.1)	1.17 (0.46-2.96), p = 0.75	6/45	13.3 (6.3-26)	11/23	47.8 (28-68.3)	0.23 (0.05-1.11), p = 0.07
Newly identified diabetes	12/144	8.3 (4.8-14.1)	7/129	5.4 (2.6-11)	1.12 (0.39-3.2), p = 0.83	5/45	11.1 (4.7-24.2)	11/23	47.8 (28-68.3)	0.23 (0.05-1.11), p = 0.07
Hypertension	63/152	41.4 (33.9-49.5)	55/134	41 (32.9-49.7)	0.76 (0.43-1.34), p = 0.34	16/47	34 (21.7-49)	16/25	64 (43.1-80.7)	0.34 (0.09-1.39), p = 0.13
Newly identified hypertension	31/152	20.4 (14.7-27.6)	26/134	19.4 (13.5-27)	0.99 (0.54-1.79), p = 0.96	13/47	27.7 (16.6-42.4)	11/25	44 (26.1-63.6)	0.91 (0.25-3.28), p = 0.89
Chronic kidney disease	18/144	12.5 (8-19)	14/132	10.6 (6.4-17.1)	1.33 (0.55-3.25), p = 0.53	1/46	2.2 (0.3-13.9)	0/25	0 (0-0)	-
Cardiovascular disease risk ≥ 20% over 10 years	5/142	3.5 (1.5-8.2)	6/128	4.7 (2.1-10)	0.4 (0.08-1.97), p = 0.26	1/45	2.2 (0.3-14.2)	0/23	0 (0-0)	-
At least one NCD	75/144	52.1 (43.9-60.2)	60/128	46.9 (38.2-55.7)	1.03 (0.57-1.85), p = 0.93	19/46	41.3 (28.5-55.4)	19/25	76 (55.3-89)	0.27 (0.07-1.04), p = 0.06
Multimorbidity**	28/156	17.9 (12.7-24.8)	22/135	16.3 (11-23.6)	1.02 (0.51-2.03), p = 0.96	6/47	12.8 (5.8-25.8)	11/25	44 (25.1-64.8)	0.42 (0.09-1.89), p = 0.26
Current smoker	33/156	21.2 (15.3-28.4)	41/135	30.4 (23-38.9)	0.85 (0.46-1.57), p = 0.6	0/47	0 (0-0)	1/25	4 (0.6-23.7)	0 (0-0), p = 0.00
Alcohol use	62/156	39.7 (32.3-47.7)	76/135	56.3 (47.6-64.6)	0.61 (0.36-1.02), p = 0.06	16/47	34 (22-48.5)	10/25	40 (23.2-59.5)	1.04 (0.25-4.36), p = 0.96
Obesity	31/154	20.1 (14.5-27.2)	16/135	11.9 (7.4-18.5)	1.45 (0.72-2.91), p = 0.3	4/47	8.5 (3.2-20.7)	4/47	8 (2-27.1)	2.95 (0.3-28.76), p = 0.35

*Adjusted for age and gender.

**Two or more conditions of diabetes, hypertension, chronic kidney disease, and HIV.

NCD: non-communicable disease; OR: odds ratio; CI: confidence interval
